# Haematological malignancies as disorders negatively impacting specificities of the direct agglutination and rapid rK39 strip tests as reference diagnostics for visceral leishmanisis

**DOI:** 10.1099/acmi.0.000522.v3

**Published:** 2023-04-11

**Authors:** Abd Elsalam Nail, Ikram Ahmed, Hussam Ali Osman, Abdallah el Harith

**Affiliations:** ^1^​ Department of Internal Medicine, Faculty of Medicine, Omdurman Islamic University and Tropical Diseases Teaching Hospital, Omdurman, Sudan; ^2^​ Internal medicine, Omdurman Teaching Hospital, Omdurman, Sudan; ^3^​ Khawarizmi International College, Al Shahama-Abu Dhabi Rd-Al Bahyah-Abu Dhabi, UAE; ^4^​ School of Pharmacy, Ahfad University for Women, Omdurman, Sudan

**Keywords:** visceral leishmaniasis, diagnosis, haematological malignancies, DAT, rK39, anti-leishmanials, SDS

## Abstract

**Introduction.:**

During several years of work in Sudan, we occasionally had been confronted with patients who presented clinical features highly suggestive of visceral leishmaniasis (VL) however direct agglutination test (DAT) readings that were either at the high negative or low positive titre range. Inquiries on the fate of those particular patients revealed mortality, undetermined diagnosis or that in some of them leukaemia was finally diagnosed.

**Gap statement.:**

Investigate as to what extent haematological malignancies (HMs) interfere with VL diagnosis.

**Aim.:**

Evaluate specificity of DAT version newly developed in this study wherein sodium dodecyle sulphate (SDS) was incorporated as a test sample denaturant in comparison with a standard reference wherein β-mercaptoethanol (β-ME) was used in test execution.

**Methodology.:**

Seventy plasma samples from patients with HMs were collected and tested in a primary DAT version (P-DAT). The results obtained were compared with those of the rK39 strip test as VL reference diagnostic. HM samples revealing titres higher than the start dilution (1 : 100) in P-DAT were further tested in a β-ME- and urea-modified DAT versions. The specificity of the newly developed SDS-DAT was assessed against that of β-ME-DAT and rK39 strip tests as current reference diagnostics for VL.

**Results.:**

Seven out of 70 patients with HMs scored positive outcomes (titre ≥1 : 3200) in P-DAT and four in the reference rK39 strip test. Of the seven that tested positive in P-DAT or four in the reference rK39, none reacted at titre >1 : 100 in the SDS-DAT. Significant reduction in non-specific agglutination reactions was achieved as a result in respect to the HM plasma samples (*P* value <0.05).

**Conclusion.:**

To establish desired specificity for VL diagnosis in respect to HMs and subsequently minimize or avoid serious side effects due to unjustified anti-leishmanials prescription the combined application of the SDS-DAT here described and an improved version of the rK39 for confirmation is recommended.

## Data Summary

No additional data is required to reproduce the results obtained in this work.

## Introduction

Based on more than 30 year laboratory and field evaluation, the direct agglutination (DAT) and rapid rK39 strip rapid tests are now widely accepted as reference diagnostics for visceral leishmaniasis (VL) [1, 2]. With the exception of African or American trypanosomiasis, malaria, typhoid, tuberculosis and autoimmune disorders these two procedures were reported to have almost absolute specificities for VL diagnosis. VL exclusion in such non-VL patients is also carried out microscopically or by detecting antigens or antibodies specific to these conditions. VL detection in respect to these non-VL conditions was furthermore enhanced as in the case of DAT, by incorporating reducing agents such as β-mercaptoethanol (β-ME) or urea in the test execution to eliminate non-specific reactions [3–5]. It is important to mention in this regard that despite incorporation of these agents in routine testing, we nonetheless had occasionally been confronted with cases that presented symptoms assimilating VL but DAT titres that were only marginally positive [unpublished data]. Unlike in our follow-up observations for detecting VL at early phases wherein steady increase in the agglutinating antibody levels was observed, DAT titres in those particular cases remained almost unchanged for several weeks [unpublished data]. Due to shortage in budget and lack of facility we were not able to correctly identify the nature of those disorders. Information gained from guardians and family members however revealed either mortality, failure to reach final diagnosis or positive investigation results for leukaemia.

Taking the current strategy for VL management as being most acceptable, anti-leishmanial administration in the (parasitologically) unconfirmed cases is justified by the presence of fever for durations of 2 weeks or longer, negative test outcomes for malaria, typhoid and tuberculosis and positive DAT (titre >1 : 3200) [1, 3, 4]. Similarly however as in VL fever, anaemia, splenomegaly, lymphodenopathy and weight loss are also among the most common clinical features presented in patients with haematological malignancies (HMs). A false-positive serological outcome that incorrectly leads to anti-leishmanial administration could therefore be harmful to these patients who may already be suffering from malfunctioning or impairment in the hemopoetic, cardiac, hepatic, renal or immune systems.

In this study, we intended to assess to what extent HMs negatively impact the specificity of DAT as reference diagnostic for VL. To minimize the risk involved in over diagnosis and treatment, we decided furthermore to evaluate performance of a new DAT version developed in this study by incorporating sodium dodecyle sulphate (SDS) as the test sample denaturant in comparison with the current reference wherein β-ME is used [3]. The relative specificity of this SDS-DAT was further assessed against that of the rK39 strip test in patients with HMs as another VL reference diagnostic.

## Methods

### HM patients

Plasma samples were collected from 70 patients received at the Hematology Clinics of the Radiology and Isotopes Centre (RICK) in Khartoum; 47 of those were males and 23 females with ages ranging 15–40 years. The number and type of the HMs identified in this patient’s population were seven lymphocytic leukaemia (ALL), four acute myeloid leukaemia (AML), seven chronic lymphocytic leukaemia (CLL), 16 chronic myeloid leukaemia (CML), 16 lymphoma, seven multiple myeloma (MM) and 13 myeloproliferative disorders (MPD).

The most important clinical features recorded were fever (77%), splenomegaly (55.2%), lymphadenopathy (52.9%), weight loss (49.4%), anaemia (39%) and hepatomegaly (21.8%). None of those 70 patients had either a recent or past history of VL or came from VL endemic area. Further interviews and investigations excluded the occurrence of malaria, typhoid, tuberculosis, African trypanosomiasis or autoimmune disorders. All plasma samples collected from the 70 HM patients were continuously stored at −20 °C.

### VL patients

Serum or plasma samples (stored at −20 °C) from our serum bank collected from 18 confirmed VL patients referred from various hospitals in Khartoum State, 12 of whom were males and 6 females. All 18 VL patients had fever for durations >2 weeks, splenomegaly, anaemia, weight loss and all of them had also positive DAT titres (1 : 3200->1 : 102400).

### P-DAT

In this initial DAT version, harvested promastigotes of *Leishmania donovani* were subjected to trypsin treatment for 45 min at 37 °C as described in detail earlier [5]. Following several times washing in Lock’s solution and centrifugation at 4 °C, the parasites were then stained with Coomassie Brilliant Blue and re-suspended at 10^8^ promastigotes/ml in normal saline supplemented with 1.2 % (v/v) formaldehyde. The ready-for-use antigen was continuously stored at 4 °C.

The test was executed by performing twofold serial dilutions of the test sample in normal saline to which Fetal Calf Serum (FCS) was added at 1.0 % (v/v) starting at 1 : 100 up to 1 : 102 400. After addition of the antigen, the test was incubated for 18 h at an air-conditioned laboratory temperature (23–26 °C) and the results were read and expressed as titres.

### β-ME DAT

Processing of the antigen was as described in details in a previous report [3]. After harvest, *L. donovani* promastigotes were repeatedly washed in Lock’s solution. Thereafter re-suspended at a concentration of 10^8^ ml^−1^ in Lock’s solution to which 0.8 % β-ME (v/v) was previously added and incubated for 45 min at 37 °C. Following washing again in Lock’s solution, centrifugation and staining with Coomassie Brilliant Blue, the stained and β-ME treated promastigotes were finally suspended at 10^5^ promastigotes/ml citrate/saline medium preserved with formaldehyde at 1.2 % (v/v) concentration.

The test was executed also according to the procedures described [3]. To determine the titre, samples were tested in twofold serial dilutions starting at 1 : 100 in a diluent containing 0.15 M sodium chloride and 0.2 % (w/v) gelatin supplemented with 0.2 M β-ME. A titre of 1 : 3200 was taken as cutoff for VL.

### Urea DAT

Processing of the antigen was the same as for the β-ME modified version [3]. Differently however, was the procedure for the test execution substituting the previously mentioned reducing agent (β-ME) by urea at a concentration of 0.1 M. Incubation and reading of test outcome were as for the β-ME-DAT [3, 6].

### SDS-DAT

To further enhance specificity for VL in DAT, performance of sodium dodecyle sulphate (CH_3_ (CH_2_)_11_ OSO_3_Na) (SDS) was evaluated as a substitute for the reducing agents β-ME and urea. Based on results gained from previous experiments [unpublished data], two concentrations of 0.09 mM or 0.045 mM in a sample diluent containing NaCL (0.15 M), CaCl2 (0.02 M), KCl (0.05 M) and NaHCO3(0.05M) were compared. Better efficiency for discrimination versus non-VL conditions was revealed by the former and therefore used to compare against results obtained with β-ME- and urea-modified versions as well as with the rK39 reference. SDS concentrations higher than those mentioned above resulted in antigen discolouring and thus invalidity of the test outcome.

In all four DAT versions described above; agglutination reactions were further classified according to titre range as strong (≥1 : 51200), moderate (1 : 12800-1 : 25600), weak (1 : 3200-1 : 6400) or negative (≤1 : 100-1 : 1600).

### rK39 rapid strip test

The test (IT Leish, Ref 710 124, Bio-Rad) was executed as instructed using 20 µl serum or plasma samples. The test reading was done after 10–15 min incubation at an air-conditioned laboratory temperature (23–26 °C) and was considered positive when in both of the control and test bands purple colour developed. Based on colour intensity, reactions were further classified as strong positive (dark), moderately positive (relatively less dark) or weak (faint). Complete absence of colour in the test band indicated negative reaction.

## Ethical approval

The study was approved by the Educational Development Centre (EDC) of the Sudan Medical Specialization, Internal Medicine, the Ministry of Health Research Department and the administration of the Radiology and the Isotopes Centre in Khartoum (RICK) (Ethical approval: TDTH Reference B/50/1). Informed consent was obtained from the patient or patient’s guardian before start of the study. Informed patient’s consent was not applicable for the 18 VL cases as inclusion of their results represent only retrospective analysis of routine data. All of the collected samples remained anonymous.

## Data analysis

The statistical analysis was done using SPSS computer software version 22.

Pair *T*-test was used to calculate significance of SDS-DAT specificity as compared to P-DAT, β-ME-DAT and Urea-DAT in respect to plasma samples from patients with HMs. *P* values <0.05 were considered to be significant. Oneway ANOVA test was used to compare between the sensitivities of SDS-DAT, β-ME-DAT and rK-39 strip test for identification of VL cases.

## Results

Out of the 70 samples collected from patients with HMs and tested in P-DAT, 16 scored titres >1 : 200, seven of whom were at the positive range (≥1 : 3200) (Table 1). Better performance was evidenced by the reference rK39, testing positive in only four that were negative in P-DAT. Despite improved specificity, still one of those 11 patients (in P-DAT and rK39) tested also positive in either the β-ME- or urea- modified DAT version (Table 2). Even though at lower reaction levels, agglutination reactions remained still measurable at 1 : 200-1 : 1600 titre range in 13 of the 70 HM patients with both β-ME- and urea-DAT versions. In none of 16 (P-DAT), 13 (β-ME-DAT or 13 (urea-DAT) HM patients who reacted at 1 : 200–12800 titre range or of 4 as positives in rK39, titres higher than the start dilution (1 : 100) were recorded in the SDS-DAT (*P* value <0.05) (Table 2). SDS-DAT revealed therefore significantly higher specificity versus plasma samples from HM patients than P-DAT, β-ME-DAT or urea-DAT (*P*-value <0.05) (Pair *T*-test).

**Table 1.** Outcome of a primary direct agglutination test (P-DAT) in comparison with that of rK39 strip test as reference diagnostic for visceral leishmaniasis (VL) in 70 patients with haematological malignancies (HMs)

Classification of rK39 reactions	No. of samples	P-DAT titre readings* ≤1 : 100 1 : 200 1 : 400 1 : 800 1 : 1600 1 : 3200 1 : 6400						
Negative	66	54	3	1	2	3	1	6
Weakly positive	1	1	0	0	0	0	0	0
Moderately positive	3	2	0	0	0	0	0	1
Strong positive	0	0	0	0	0	0	0	0

*Cutoff titre for P-DAT is 1 : 3200.

**Table 2.** Titre readings obtained with β-mercaptoethanol (β-ME-DAT)-, urea (urea-DAT)- and sodium dodecyle sulphate-modified direct agglutination test (SDS-DAT) versions in 16 patients with haematological malignancies (HMs) scoring titres >1 : 200 in a primary DAT (P-DAT)

Titre reading*	DAT version P-DAT β-ME-DAT Urea-DAT SDS-DAT			
1 : 200	3	10	11	0
1 : 400	1	1	1	0
1 : 800	2	0	1	0
1 : 1600	3	2	0	0
1 : 3200	1	0	1	0
1 : 6400	6	0	0	0
1 : 12 800	0	1	0	0

*Cutoff titre for all four DAT versions is 1 : 3200.

In agreement were the results based on positive or negative outcome obtained with SDS-DAT or β-ME-DAT in all 18 VL patients (Table 3). Of ten VL patients who showed strong agglutination intensities in SDS-DAT, eight did similarly in the reference rK39. Among 16 who strongly tested in SDS-DAT, ten did equally so in β-ME-DAT. Unlike however with β-ME-DAT or SDS-DAT, four of the 18 VL patients tested unexpectedly weakly positive (2) or even negative (2) with the reference rK39. SDS-DAT and β-ME-DAT showed therefore significantly higher reliabilities for VL detection than rK39 strip test (respective *P* values of 0,029 and 0,027).

**Table 3.** Outcome of a newly developed sodium dodecyle sulphate-modified direct agglutination test (SDS-DAT) in comparison with that of a β-mercaptoethanol equivalent (β-ME-DAT) and an rK39 strip test as reference diagnostics for visceral leishmaniasis (VL) in 18 confirmed cases

DAT version	Titre range*	Classification of reactions with rK39 strip test			
		Strong	Moderate	Weak	Negative
β-ME-DAT	1 : 3200- 1 : 6400	1	0	0	1
	1 : 12800-1 : 25 600	3	0	2	1
	>1 : 51 200	8	2	0	0
SDS-DAT	1 : 3200- 1 : 6400	0	0	0	0
	1 : 12800-1 : 25 600	0	0	2	0
	>1 : 51 200	12	2	0	2

*Cutoff titre for β-ME-DAT and SDS-DAT.

Similarly as with urea, however differently than with its β-ME equivalent, use of SDS as serum denaturant at such a low concentration seemed equally practical, economical and safe for routine and mass VL detection.

## Discussion

If promptly combined with administration of anti-leishmanials, VL detection at the early phase is considered the best strategy for preclusion of resistance and relapse. Equally important however is that the anti-leishmanial prescription should adequately be justified in particular with respect to patients who are already also suffering from existing serious disorders such as HMs. Cardiac arrhythmia, pancreatitis, nephrotoxicity, severe vomiting, diarrhoea and gastrointestinal toxicity are among the most common side effects of anti-leishmanial therapy [2, 7].

Despite excellent diagnostic reliability reported for both β-ME-DAT and rK39, cross reactions with infectious and non-infectious diseases were occasionally or frequently reported depending on the study area and population involved [5, 8, 9]. In our opinion, absolute specificity still remains a future objective for these two diagnostics as well as for others that are currently undergoing development. This is the first study in which specificities of the VL reference diagnostics are rigorously assessed by challenging them against plasma samples from such a large number (70) of patients diagnosed with seven different types of HMs. The elevated immunoglobulin G levels derived not only from the B-cells of these patients but also from their tumour cells is expected to increase the possibility for cross reactions with other antigens such as these of *Leishmania*.

Because of their invasive nature, lower sensitivity and the adequate expertise required to demonstrate the causative amastigotes, use of parasitological methods is rendered less important in routine and mass screening of VL. Following the current protocol for VL management, patients with typical disease symptoms and positive DAT and or rK39 outcome should be considered for treatment [1, 2, 10–13]. In this study group of HM patients, symptoms highly similar to VL were recorded in 50–70 % of them. Among those 70 HM patients, seven also tested positive in P-DAT and four in the rK39 reference. Based on the VL management strategy recommended, all of the 11 suspects should have theoretically been put on treatment with first-line anti-leishmanial drugs running thus unfortunately the risk that some or all of them could acquire at least one of the side effects above mentioned. The chances for such a risk is rendered even higher when considering the possibility of existing malfunction or impairment in the vital organs or systems of those patients. Additional side effects could also develop should failure to achieve favourable response to the first-line anti-leishmanial wrongly be interpreted as resistance. As a consequence, a second-line treatment regimen may be started leading even to a life-threatening situation.

In order to minimize or avoid over-diagnosis, the simultaneous application of β-ME-DAT and rk39 is recommended [1, 2]. In this study, 4 of the 70 HM patients tested positive in rK39, 3 of whom scored unexpectedly unambiguously negative titres (≤1 : 100) in β-ME-DAT. Based on this finding and on others previously reported, we strongly believe that only due to the introduction of β-ME and later on urea to reduce non-specific reactions, specificity comparable to that of the rK39 reference was achieved in the DAT [3, 6]. Together with the latter, the two tests were recommended therefore as complementary for VL diagnosis in the undetermined cases. Unfortunately our efforts to achieve similar reduction in non-specific reactions as regards the rK39 reference were less successful (unpublished observation). Our conclusion is that because of the presence of these non-specific reactions, the DAT as here further improved with SDS evidenced better diagnostic reliability than the current rK39 version even when plasma samples from HM patients were included.

Concomitant with improvement in specificity, the incorporation of SDS also enhanced the sensitivity in DAT (Table 3). Unlike with rK39, all 18 VL samples scored positive titres in the SDS-DAT, 16(88.9 %) of whom showed even strong agglutination reactions. Matching high sensitivity levels were reported earlier also with a multi-epitope development of the reference rK39 (rK28) in two different VL endemic areas in Sudan [9, 14]. It is highly likely therefore that the simultaneous SDS-DAT/ rK28 application could provide a better approach to the specific VL diagnosis in Sudan.

Based on merits here and previously enumerated included high diagnostic reliability, antigen stability under adverse conditions and the required safety for test execution, the new SDS-DAT is expected to comply with most of the criteria needed for an appropriate and safe management of VL [13, 15].

## Conclusion

As in VL, symptoms such as fever, splenomegaly, lymphadenopathy, weight loss, anaemia and hepatomegaly are also among the most common clinical features in HMs. In this study, 11 out of 70 patients with HM disorders revealed in addition to these symptoms positive outcomes in two VL reference diagnostics namely the DAT and rK39 strip test. The decision to prescribe anti-leishmanials for the (parasitologically) unconfirmed cases of VL should therefore adequately be justified in order to avoid serious side effects. In this study, we have assessed as to what extent disorders such as HMs can negatively impact reliabilities of the DAT and rK39. To minimize the risk involved, a new DAT version was developed by incorporating SDS (SDS-DAT) as sample denaturant in the test execution. The relative diagnostic reliability of this SDS-DAT was assessed against that of βM-DAT and rK39 strip tests as reference VL diagnostics.
